# Prevention and control of noncommunicable diseases: lessons from the HIV experience

**DOI:** 10.2471/BLT.18.216820

**Published:** 2019-02-01

**Authors:** Seye Abimbola, Emma Thomas, Stephen Jan, Barbara McPake, Kremlin Wickramasinghe, Brian Oldenburg

**Affiliations:** a School of Public Health, University of Sydney, Sydney, Australia.; b Melbourne School of Population and Global Health, Level 2, Lincoln Square North, University of Melbourne, 3052 Melbourne, Australia.; c The George Institute for Global Health, University of New South Wales, Sydney, Australia.; d World Health Organization European Office for the Prevention and Control of NCDs, Moscow, Russian Federation.

In many low- and middle-income countries, the challenges of scaling up successful localized projects to achieve national coverage are well recognized.^1^ However, because of the widely acknowledged success of national efforts to scale up interventions to prevent and control human immunodeficiency virus (HIV) infection, the disease is now largely managed as a chronic condition. The shift means that lessons from the HIV experience may be transferable to the rollout and scale-up of effective interventions for noncommunicable diseases in low- and middle-income countries.^2^ The scale-up of noncommunicable disease interventions is particularly important because coverage is still modest and the evidence base for implementation in low- and middle-income countries remains very limited.^3^

WHO’s best buys for reducing noncommunicable diseases in low-resource settings suggest several such interventions. The interventions include measures to improve tobacco control, increasing public awareness of the health benefits of physical activity, multidrug therapy for people at high risk of cardiovascular disease and the screening and treatment of cervical cancer.^3^ Low- and middle-income countries need to rapidly disseminate and implement effective noncommunicable disease interventions at a national scale, often in contexts of low resources and capacity. The risk of premature death (that is, of people younger than 70 years) from the four major noncommunicable diseases (cardiovascular diseases, cancers, chronic respiratory diseases and diabetes) decreased by 25.4% in high-income countries and by 24.4% in upper-middle-income countries between 2000 and 2015. However, such mortality only reduced by 7.8% in lower-middle-income countries and increased by 6.0% in low-income countries.^4^

Lack of context-specific evidence to underpin adaptation and implementation of best buys in low-income and lower-middle-income countries exacerbates this difference, as the bulk of research evidence on innovations to address noncommunicable diseases is from high-income countries.^3^ Despite limited evidence and capacity for adaptation of these best-buy interventions in low-resource settings, policy-makers and implementers in many low- and middle-income countries are expected to adapt and implement interventions from high-income countries and to move from pilot to implementation at scale. Nonetheless, some of the models of service delivery that have enabled the delivery of long-term care at scale may be transferable from HIV intervention scale-up to noncommunicable disease interventions. Lessons from the HIV experience, relevant to governance, financing, human resources, service delivery, products and technologies, information systems and community mobilization, and their potential applications to noncommunicable diseases are summarized in Table 1.

**Table 1:** Application of HIV lessons in low- and middle-income countries to noncommunicable disease intervention scale-up

Barriers to scale-up	Solutions from the HIV field	Application to noncommunicable disease interventions
Governance	• Performance-based financing • Global and national target-setting	HIV field used global targets and commitments, such as the MDGs to engage political action. This is now happening for noncommunicable disease prevention and control, with the UN High-Level meetings and the SDGs.
Financing	• Removal of user fees • Price negotiations	The HIV experience suggests that public-private partnerships may be important in ensuring equitable access to treatment, thereby facilitating scale up on the most resource-poor settings. Such arrangements, involving preferential pricing, may not be necessary for many cardiovascular disease treatments that are largely off patent, but potentially relevant to new generations of cancer treatments.
Human resources	• Task-shifting to and task-sharing with community health workers • Expert patients and peer supporters	Transferring ART care from doctors to nurses has been shown to be an effective and cost–effective strategy within HIV treatment.^5^ For noncommunicable disease interventions historically delivered by health professions (such as risk-factor screening) can be delivered by peer-leaders and/or community health workers.
Service delivery	• Decentralised services • Adherence support • Infrastructure repairs and renovations to health-care facilities	Examples for noncommunicable diseases include modifications in service delivery such as decentralized community–based noncommunicable disease care and the use of community health workers for medication adherence support.
Products and technologies	• Supply chain and procurement systems development • Mobile health interventions	Mobile health delivery systems and short messaging services are enabling large-scale risk detection, behaviour change interventions, and monitoring and evaluation efforts in noncommunicable disease.
Information systems	• On-site and electronic medical records • Harmonization of information platforms	The HIV experience involved developing parallel information systems that were subsequently integrated into national health information systems. There are now efforts to similarly integrate noncommunicable disease information into national systems alongside HIV information, including with the use of electronic platforms.^6^
Community mobilization	• Co-designed interventions and quality improvement systems	Collective community action and systems thinking is being used to co-design whole-of-community solutions for noncommunicable diseases.^7^

ART: antiretroviral therapy; HIV: human immunodeficiency virus; MDG: millennium development goal; SDG: sustainable development goal; UN: United Nations.

However, acknowledging that significant differences exist between HIV and noncommunicable diseases and that these differences influence implementation and scale-up is important. For instance, evidence for the scale up of HIV-care delivery was primarily generated and applied, and strategies for widespread implementation and scale-up were initially attempted, in low- and middle-income countries. The spread of innovation among low- and middle-income countries was therefore less constrained. Adopting interventions to prevent and control noncommunicable diseases also requires a contextual adaptation of strategies, as these were primarily developed in high-income countries. Ease of adoption and adaptation to local context is key to successful scale-up. For example, while integrated team-based care for people with complex lifelong chronic conditions is important, delivery of such care in a low-resource health system will be different to that in well-resourced health systems. 

Scale-up is not only technical; it is also political. Scale-up is about what gets on the agenda of governments, global health agencies or philanthropists. HIV was an infectious disease that high-income countries sought to keep outside their borders, hence stimulating support for scale-up of prevention and control measures in low- and middle-income countries. Upstream influences (for instance, the commercial determinants of health) that are driving the increase of noncommunicable diseases are outside the health system. HIV affected population groups whose strong identity fostered collective action, contributing to placing scale-up on the political agenda in many low- and middle-income countries. Movements such as NCDFREE and the NCD Alliance seek to build similar traction for noncommunicable diseases. Addressing noncommunicable diseases may require legal and fiscal measures that are unpopular for industries, such as taxes on tobacco, alcohol and sugar-sweetened beverages, and regulation to reduce salt in processed food.

In addition to the specific lessons gained from the HIV field, efforts to adopt lessons and innovations from the HIV experience to scale up noncommunicable disease interventions should be informed by several approaches. The first is to follow a structured approach to re-design innovations for scale. The WHO guide^1^ for scaling-up suggests that the most likely innovations have the CORRECT features, that is that they are: credible; have observable results; are relevant and address key community issues; provide a relative advantage over existing practice; are easy to install; are compatible with established norms, values and existing programmes; and are testable. The second approach is to conduct a systematic assessment of contextual drivers that affect scale-up. Systems in which population and community-based interventions are implemented are complex and unpredictable. Understanding the mechanisms of action of an intervention as well as contextual factors within a system that may either enable or constrain implementation is therefore important. Co-design, participatory design, systems thinking and realist analysis are examples^8^ of approaches that assist in understanding complex health and social systems. These approaches also contribute to identifying contextual factors such as institutional, socioeconomic and geographical factors, including inequities of access to health care and other social determinants of health. All these factors are likely to interact with interventions and influence their implementation, scale-up and outcomes. The third approach is to embed continuous improvement in the design of scalable programmes. Implementation of interventions to large populations should incorporate appropriate feedback loops and quality improvement, so programmes can be adapted and sustained over time. This approach requires a shift from the traditional evidence-based paradigm that predetermines what evidence and outcomes are required before a programme is implemented to a more adaptive and systems-based approach.^9^ For example, in collaboration with WHO, a guide on implementation research for noncommunicable disease prevention and control has recently been published.^10^ The guide includes an initial situational analysis, identifies appropriate policies or interventions, and offers guidance on adapting, piloting, implementing and evaluating the intervention, as well as scaling-up with iterative feedback. The fourth approach is to actively disseminate learnings to inform scale-up in other settings. If identified learnings were placed in an open-source repository, this could help create a generalizable evidence base from different case studies that could, over time, be augmented by new research findings in other contexts. Such data-pooling can enrich our understanding of mechanisms that drive successful scale-up and of context, which others can subsequently use for adapting and contextualizing programmes and policies to new settings. This approach has been used for many years by the International Tobacco Control Policy Evaluation Project.^11^ Transnational communities of practice, innovation platforms and other forms of professional networks have been used as vehicles for sharing implementation experience and spreading innovations in health and other policy areas, and may be similarly deployed for global noncommunicable disease prevention and control scale-up efforts.^12^^,^^13^

The last approach is to use global data monitoring, measurement and evaluation platforms. Field learning on scale-up should be progressively systematized to ensure that we can also gradually learn from the scale-up of noncommunicable disease interventions from one setting to another.^14^ While traditional research funding prioritizes time-limited trials or demonstration projects in defined populations using study designs that maximize internal validity, scale-up research requires longer-term funding of ongoing evaluation of implementation efforts. Although such research and evaluation may be led by researchers, scale-up research will rely on partnerships with practitioners, at the policy, programme and clinical levels, to facilitate application. In addition, more sustainable approaches to secure funds for scale-up research will be necessary and will require funding support from major research funding agencies, governments and WHO.

While there is much to learn from the HIV experience, noncommunicable diseases have peculiarities that may limit the transferability of learnings or require significant adaptation of such learnings. The same applies to the transferability of learnings on noncommunicable disease prevention and control between high-income and low- and middle-income countries. However, the scale-up of interventions to prevent and control noncommunicable diseases, especially in low- and lower-middle-income countries, presents an opportunity for the use of research to facilitate quick and potentially global spread of effective and innovative interventions. We therefore call for the development of research and practice platforms that allow for progressive and systematic accumulation and sharing of field learnings from scale-up efforts. These platforms will maximize learning from the experience of scale-up of HIV interventions and from the scale-up of noncommunicable disease interventions between settings.
